# Psychosocial Impact of the COVID-19 Pandemic Among Omanis with Multiple Sclerosis: Single Tertiary Center Experience

**DOI:** 10.3390/ijerph22081236

**Published:** 2025-08-07

**Authors:** Jihad Yaqoob Ali Al Kharbooshi, Abdullah Al-Asmi, Ronald Wesonga, Samir Al Adawi, Amal S. S. Al-Fahdi

**Affiliations:** 1 Department of Clinical Neurological Sciences, Western University, London Health Sciences Center, London, ON N6A 3K7, Canada; 2 Neurology Unit, Department of Medicine, College of Medicine & Health Sciences, Sultan Qaboos University, Muscat 123, Oman; 3 Department of Statistics, College of Science, Sultan Qaboos University, Muscat 123, Oman; wesonga@squ.edu.om; 4 Department of Behavioral Medicine, College of Medicine and Health Sciences, Sultan Qaboos University, Muscat 123, Oman; samir.al-adawi@fulbrightmail.org; 5 Psychosocial Unit, Department of Holistic Care, Sultan Qaboos Comprehensive Cancer Care and Research Center, University Medical City, Muscat 123, Oman; a.alfahdi@cccrc.gov.om

**Keywords:** multiple sclerosis, disease modifying therapies, COVID-19 pandemic, psychological effects, mental well-being, Oman

## Abstract

(1) Background: The COVID-19 pandemic presented unprecedented challenges for people with multiple sclerosis (PwMS) in Oman, necessitating targeted healthcare planning and patient support. This study aimed to investigate the impact of COVID-19 on MS management and disease course, incidence, and outcomes of COVID-19, psychosocial and mental health effects of the pandemic, and demographic and clinical predictors of the effects related to COVID-19 among Omani PwMS. (2) Methods: This cross-sectional study was conducted from January to April 2021. Adult (18–60 years) Omani PwMS completed a structured interview along with the Expanded Disability Status Scale (EDSS) and World Health Organization Well-being Index (WHO-5). Clinical data on relapses and disease-modifying therapies and adherence were analyzed. The data was statistically analyzed. (3) Results: Of 104 PwMS (73.1% female), 22.1% contracted COVID-19, with fatigue being the most reported symptom (87%). Female sex (*p* = 0.042), younger age (18–34 vs. 35–45 years; *p* = 0.014), diagnosis of COVID-19 (*p* = 0.037), and low current mental well-being scores (*p* = 0.021) predicted greater COVID-19-related effects. (4) Conclusion: These findings highlight the need to study the mental resilience of this subgroup of PwMS and provide them with targeted support during crises.

## 1. Introduction

On 11 March 2020, the World Health Organization (WHO) declared COVID-19 a global pandemic [1]. By 30 April 2021, Oman had recorded 193,253 confirmed cases, 191,213 recoveries, and 2040 deaths. With an estimated MS prevalence of 15.9 per 100,000, Oman is classified as a medium risk zone for the disease [2].

As healthcare systems around the world struggled to manage the pandemic, individuals with chronic health conditions faced significant barriers to accessing physician appointments, diagnostic tests, prescriptions, and hospital care. This challenge was particularly pronounced for people with multiple sclerosis (PwMS), a progressive neurological disease that affects primarily younger adults and significantly affects their quality of life.

A major challenge for PwMS during the COVID-19 pandemic was the management of disease-modifying therapies (DMTs), which often cause immunosuppression and require strict monitoring through neuroimaging and blood tests. DMTs vary in mechanisms, dosing, administration and monitoring, each carrying distinct risks related to COVID-19. For example, alemtuzumab significantly suppresses the immune system, increasing susceptibility to COVID-19 infection and related complications, while beta-based interferon-based treatments are immunomodulatory and carry a relatively lower risk [3]. As a result, recommendations were made to delay more immunosuppressive DMT (e.g., alemtuzumab) and extend the intervals between doses of B cell-depleting therapies [4]. Clinicians managed PwMS on an individualized basis, considering age, comorbidities, MS severity, relapse frequency, and DMT type. It became clear that those on immunosuppressive regimens, with severe disability or with a longer disease duration, were particularly vulnerable to severe COVID-19 infection [4].

The COVID-19 pandemic and its associated restrictions also had profound psychosocial effects on both the general population and the clinical groups [5,6,7]. Toward the beginning of the pandemic, Wang et al. (2020) reported that more than half of their Chinese participants had poor psychosocial outcomes, 16.5% and 28.8% experienced moderate-to-severe depression and anxiety, respectively [6]. Even before the pandemic, PwMS were known to have an increased risk for psychosocial problems. For instance, in our own pre-pandemic study in 2015, 51% of Omani PwMS had reported symptoms of poor well-being [8].

During the pandemic, Oman’s Ministry of Health (MOH) advised people with chronic diseases to adopt stricter self-protective measures, including self-isolation to reduce the risk of contracting the virus [9]. While this was a lifesaving measure, it potentially exacerbated psychosocial challenges, particularly for younger PwMS [10]. However, the psychosocial impact of the pandemic on Omani PwMS is yet to be investigated.

In light of these challenges, this study aimed to: (i) evaluate the impact of COVID-19 on the management of MS and disease progression, (ii) investigate the incidence and outcomes of COVID-19 infection among PwMS, (iii) assess the psychosocial and mental health effects of the pandemic on PwMS, and (iv) identify the demographic and clinical predictors of COVID-19-related effects.

## 2. Materials and Methods

This hospital-based cross-sectional study on Omani PwMS at Sultan Qaboos University Hospital (SQUH) collected data between January and April 2021. Data was collected from potential participants via telephone interviews, after obtaining their informed verbal consent, using a structured questionnaire. A research assistant, trained and supervised for consistency and accuracy, conducted the interviews. The Hospital’s electronic medical records were reviewed to ensure completeness and precision of the diagnosis based on the revised McDonald 2017 diagnostic criteria [11]. COVID-19 diagnosis was confirmed by SARS-CoV-2 PCR testing on nasopharyngeal swabs.

Inclusion criteria were as follows: Omani PwMS aged 18 years diagnosed with relapsing-remitting MS (RRMS), secondary progressive MS (SPMS), or primary progressive MS (PPMS) who met the McDonald 2017 revised diagnostic criteria and attended the SQUH Neurology Clinic during the study period [11].

The study excluded non-Omani nationals, PwMS under 18 years of age, those diagnosed with neurological conditions other than MS, patients with severe comorbidities (e.g., advanced cardiovascular disease, cancer, or uncontrolled diabetes), and patients unable to provide informed consent due to cognitive impairment or legal restrictions.

For outcome measures, participants were classified according to sex, age, duration of MS in years, and other demographic and disease-specific variables. The Expanded Disability Status Scale (EDSS) was used to assess disability severity, scored as follows: none (0), slight (1–1.5), minimal (2–2.5), moderate (3–4.5), and severe (≥5). The DMTs used were classified by mode of administration: (1) oral DMT (teriflunomide, fingolimod, dimethyl fumarate), (2) oral reconstitution therapy (cladribine), (3) infusion DMT (ocrelizumab, rituximab and natalizumab), and (4) injectable DMT (interferons and glatiramer acetate).

The perceptions of the physical and psychosocial impact of the pandemic were collected, including details of the COVID-19 infection, symptoms, and severity. These included whether participants experienced MS relapses during the pandemic, received an intravenous corticosteroid (methylprednisolone) for relapse treatment, had difficulty obtaining prescriptions, continued to take their DMT regimen, or experienced disruptions in accessing neurologist or MRI appointments. These elements were informed by the relevant literature [12,13,14], with local linguistic experts and neurologists refining the survey for relevance to Omani MS patients. The finalized items, presented in Table 1, were evaluated for internal consistency, which was found to be satisfactory. Participants who tested positive for COVID-19 were asked about their experiences with symptoms defined by the WHO (2020), including fever, shortness of breath, chest pain, dry cough, sore throat, fatigue, headache, loss of taste, diarrhea, and redness of the eyes [1]. The study also explored the impact of COVID-19 infection on MS, treatment settings (home or hospital), and recovery status.

Mental well-being was assessed using the World Health Organization Well-being Index (WHO-5) [14]. WHO-5 consists of five positive statements evaluated based on feelings over the past two weeks. Examples include: “I have felt cheerful and in good spirits;” “I have felt calm and relaxed;” “I have felt active and vigorous;” “I woke up feeling fresh and rested;” “My daily life has been filled with things that interest me” [14]. Responses are scored on a Likert scale of 0 to 5, with the overall score ranging from 0 to 25. This score is then multiplied by 4 to derive the percentage score, where 0% represents ‘worst well-being’, and 100% ‘best well-being’ [15]. WHO-5 has been widely used in Arabic-speaking populations with adequate psychometric properties [16]. We employed the Cronbach alpha test to assess the internal consistency and reliability of measurements of the WHO-5 scale. The test indicated that 93% of the observed variance was attributable to true score variance.

Statistical analysis was performed using R version 4.2.2 [17]. Descriptive statistics were used to summarize the data; continuous variables were reported as mean with standard deviation (±) and categorical variables as frequencies and percentages. Where required, 95% CI was calculated to indicate precision levels. Chi-square tests examined relationships between categorical variables (e.g., demographic and clinical characteristics) and the reported effects of COVID-19. For variables that showed significant associations, a logistic regression analysis was performed to identify independent factors. Univariate and multivariate logistic regression models were used to explore potential confounders. Variables with substantial missing data were excluded. Key assumptions (linearity, influential values, and multicollinearity) were adequately tested before the univariate and multivariate logistic regression models were fitted. Fisher’s exact test was applied when the sample sizes were small (e.g., male−female differences in COVID-19-affected PwMS). All statistical tests were two-sided; significance level was set at *p* < 0.05.

## 3. Results

A total of 104 Omani PwMS participated in this survey. Their average age was 39 years, and the majority (73.1%) were women. The mean EDSS score was 2. The participants’ general MS care was mostly unaffected during the pandemic, with 97% reporting no problems getting neurologist appointments. However, 24% reported that their MRI appointments were affected and 16.3% reported experiencing MS relapses during the pandemic period (Table 1).

We compared the demographic and clinical information between COVID-19-infected participants (n = 23) and uninfected PwMS (n = 81). No significant between-group differences were found in age, disease duration, EDSS scores, relapse rates, or utilization of DMTs during the pandemic. However, the proportion of males was significantly higher in the COVID-19-infected group (47.8%) than in the non-infected group (21.0%; *p* = 0.022). This suggested a potential association between male sex and increased risk of COVID-19 infection within this population, supporting the rationale for the analyses presented in Table 2 and Table 3.

The vast majority of PwMS (90.4%) continued taking their prescribed DMT during the pandemic. PwMS taking oral DMT (teriflunomide, fingolimod, dimethyl fumarate) were more likely to contract COVID-19 (31.6%) compared to those of other types of DMT such as infusion DMT (ocrelizumab, rituximab, natalizumab) (22.0%) (Table 1). Regarding specific DMT, 5 of 23 (21.7%) PwMS who were taking anti-CD20 infusions and 4 of 18 (23.5%) PwMS on natalizumab contracted COVID-19. None of those who received injectable DMT (interferons and glatiramer acetate) or oral immune reconstitution therapy (IRT) got infected. The rate of MS relapse among COVID-19-infected individuals was higher than those not infected (Table 1).

Table 2 summarizes the prevalence of COVID-19 symptoms among the infected 23 Omani PwMS (men: n = 11; women: n = 12). The most reported COVID-19-related symptoms were fatigue (87.0%), loss of taste (78.3%), fever (73.9%), and headache (69.6%). There were no significant differences in the prevalence of symptoms between sexes.

Most COVID-19 cases (91.3%) were managed at home, with 73.9% patients achieving full recovery, with no significant difference between sexes. Regarding the impact of COVID-19 on MS, 73.9% of our participants did not experience lasting effects. One male patient (4.3%) reported worsening of MS symptoms (Table 2). Although men were less likely to report no lasting effects of COVID-19 on multiple sclerosis (MS) or worsening MS symptoms than women, differences were not statistically significant, based on Fisher’s exact test. The small sample size limited the ability to detect significant differences.

Table 3 shows the analysis of COVID-19-related worries among our participants during the pandemic period. Responses were categorized into three domains: concerns about COVID-19 infection, healthcare access, and MS relapse. Frequently reported concerns were being worried “all the time” about healthcare access (51.9%), becoming infected with COVID-19 (46.2%), and experiencing an MS relapse (60.7%). When stratified by sex, no significant differences were found across any of the domains. Although a larger proportion of men (64.3%) than women (39.5%) reported being worried “all the time” about COVID-19 infection, the overall distribution did not differ significantly (χ^2^ = 5.982, *p* = 0.200). No significant sex-based differences were observed for concerns about healthcare access (χ^2^ = 3.534, *p* = 0.472) or MS relapse (χ^2^ = 6.282, *p* = 0.099). Overall, male and female PwMS reported similar levels of pandemic-related concern (Table 3).

The details of the psychosocial effects of the COVID-19 pandemic among Omani PwMS are presented in Table 4. The table shows descriptive distributions of the binary dependent variable (COVID-19 effect versus no effect) and factors with significant associations with the effects, as assessed using univariate and multivariate logistic regression. Although chest pain and MS relapse were not significantly associated with reported effects in the multivariate model, chest pain bordered on significance (*p* = 0.055). Lower scores on current mental well-being were independently associated with increased odds of reporting pandemic impact (adjusted OR = 0.88; 95% CI: 0.79–0.98; *p* = 0.021) (Table 4).

Table 4 also shows that women were more likely to report pandemic-related effects than men (adjusted OR = 3.17; 95% CI: 1.04–9.61; *p* = 0.042). Individuals aged 35–45 years were significantly less likely than 18–34-year-olds to do so (adjusted OR: 0.18; 95% CI: 0.05–0.71; *p* = 0.014). Although a confirmed COVID-19 diagnosis was non-significant in univariate analysis, it emerged as a strong predictor in the multivariate model (adjusted OR = 5.76; 95% CI: 1.11–29.85; *p* = 0.037).

**Figure 1:**
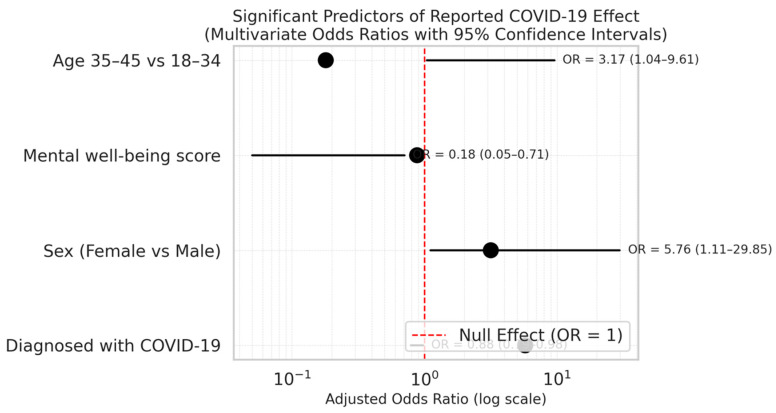
Diagrammatic representation of significant predictors for reporting COVID-19 pandemic effects among Omanis with multiple sclerosis (n = 104).

Figure 1 is a forest plot presenting a summary of adjusted odds ratios (ORs) with 95% confidence intervals for significant predictors of reported COVID-19 effects. The vertical red dashed line at OR = 1 denotes the null effect. Variables with confidence intervals not crossing this line are statistically significant. Below are the key highlights:

- Being female and diagnosed with COVID-19 were associated with significantly higher odds of reporting pandemic-related effects.
- Middle-aged individuals (35–45 years) had significantly lower odds of reporting effects compared to the youngest age group.
- Lower mental well-being scores were independently linked to greater odds of reporting pandemic impact.

## 4. Discussion

Of the 104 Omani PwMS in this cohort, 23 (22.1%) contracted COVID-19, but they experienced mild symptoms. This prevalence is significantly higher than that reported in 2022 from another tertiary center in Oman, where only 2.5% of 240 PwMS tested positive for COVID-19 [18]. Our figures are also significantly higher than the prevalence of 4.3% in the general population of Oman at the end of April 2021 [19]. However, the prevalence of COVID-19 in our participants was consistent with those reported by MS centers in other parts of the world. For example, a prevalence of around 25% among 232 PwMS was found in Italy by April 2020 [20]. In the Netherlands, the prevalence was as high as 50% among 86 PwMS [21]. A multicenter French study reported approximately 28% COVID-19 prevalence among 399 PwMS [22]. Such large variations between studies could have been due to multiple factors, including the timing of data collection, patient selection, reporting method, overall infection rate in the population, and the timing of COVID-19 vaccination rollouts.

The most common symptoms of COVID-19 in our cohort were fatigue (87.0%), taste loss (78.3%), and fever (73.9%). On the contrary, fever was the most common symptom of COVID-19 (78.4%) in a study covering Oman, Kuwait, and the United Arab Emirates (UAE) [18]. Asthenia was the most common symptom (83.6%) among PwMS in the French study mentioned above [21]. In a recent retrospective study by Zeineddine, based on the MENACTRIMS registry (Middle East North Africa Committee for Treatment and Research in Multiple Sclerosis), in the MENA region, which reviewed 600 PwMS with COVID-19, the three most commonly reported symptoms were fever, cough, and anosmia/ageusia [23].

In Poland, fever (56.2%) and fatigue (40.5%) were the main symptoms [24]. In a 2024 survey in the United States (U.S.), PwMS recalled experiencing significantly increased levels of physical, mental, and emotional fatigue during the COVID-19 lockdown period, and that these symptoms remained higher than pre-lockdown levels even after the restrictions were lifted [25].

Most of our PwMS infected with COVID-19 experienced mild symptoms, mainly managed their illness at home, and made a full recovery with no long-lasting effects of the disease. One reason for this may have been the proactive steps taken by the Ministry of Health to maintain essential healthcare services including support to chronic patients during the pandemic [19]. Outpatient services prioritized emergency care and chronic critical conditions, including MS. Many polyclinics established hotlines, encouraging patients with chronic diseases to use WhatsApp and phone calls to inquire about the availability of medications.

Systematic scheduling of monthly medications and follow-up appointments enabled 97% of our participants to report ‘no difficulty’ in accessing neurologist appointments. MRI appointments were affected more, with 19.2% reporting delays, though less compared to a Latin American cohort where 33% of PwMS reported postponed MRIs and 69% experienced delays in initiating their DMTs [26]. In New York City, there were widespread postponements of medical appointments, laboratory tests, and MRIs [27].

### 4.1. DMT and COVID-19

Most PwMS in our study continued their DMT regimen during the pandemic. Those taking oral DMT (teriflunomide, fingolimod, dimethyl fumarate) were more likely to contract COVID-19 (31.6%) compared to those on infusion DMTs (ocrelizumab, rituximab, natalizumab) (22.0%). Among specific DMTs, 21.7% of patients receiving anti-CD20 infusions contracted COVID-19, while 22.2% of those taking natalizumab did. Interestingly, none of the patients injectable DMT (interferons, glatiramer acetate) or oral IRT contracted COVID-19.

The medication trends among our PwMS with COVID-19 during the pandemic were similar to those in other GCC countries. For example, Saudi Arabian PwMS with COVID-19 mostly received injectable interferons and fingolimod [28]. A multicenter study across Oman, Kuwait, and UAE found fingolimod and ocrelizumab to be the preferred DMTs [18]. Ocrelizumab was also the most used DMT in a cohort of PwMS in the U.S. [29].

A recent longitudinal review found some DMTs—including fingolimod and anti-CD20 therapies such as ocrelizumab and rituximab—to be associated with increased immunosuppressive risks, potentially increasing vulnerability to COVID-19 infection [30].

### 4.2. COVID-19 Effects

Our participants experienced significant COVID-19-related anxiety. Moderate-to-severe anxiety was also observed among PwMS in Spain, Saudi Arabia, and Iran [31,32,33].

Women comprised 73.1% of our participants. This is slightly higher than the female-to-male ratio reported in Omani epidemiological data (2.17:1, or 68.5%), as well as the global average (69%) and the EMR average (66%) [2,34]. The higher female proportion in our cohort may be due to single-center sampling, greater willingness of women to participate, or the small sample size.

In our study, women were significantly more likely to report the effects of the pandemic. A similar trend was observed in the general populations of Oman and the U.S. [35,36]. Further, our youngest participants [18–34 years] were significantly more likely to report the effects compared to older PwMS. This is corroborated by other studies, which have attributed this greater vulnerability of younger PwMS and women to increased psychological distress, health consciousness, disruption to daily life, caregiving stress (especially in women), and social or economic instability [37,38].

While our study did not specifically investigate targeted interventions, the potential of enhancing resilience is essential among PwMS and remains a key priority. These potential strategies include psychosocial interventions, mindfulness-based stress reduction, peer support programs, and psychoeducation. To date, in Oman, access to such services for PwMS in Oman remains limited and is mostly confined to tertiary care centers, which are often located in urban areas. Expanding community outreach programs and involving primary health care providers would be essential for improving access and support. Furthermore, a multidisciplinary approach—integrating neurologists, psychologists, social workers, and other allied professionals—could offer a more comprehensive approach and enhance the effectiveness and reach of care.

### 4.3. Mortality

There was no report of mortality associated with COVID-19 in our sample. However, deaths have been reported from other parts of the world. A large study in Sweden (n = 17,692 PwMS), based on data from early 2020, reported a mortality rate of 0.38/100,000 person-years, vs. 0.15 in the general population [39]. A meta-analysis of 18 studies (5634 PwMS) reported a pooled COVID-19 mortality rate of 1.97%, with higher lethality linked to age, comorbidities, progressive MS, and anti-CD20 therapies [40]. Another meta-analysis of 14 pre-vaccine studies in pre-vaccine settings found a 1.97% mortality rate among PwMS with COVID-19, with no rise in relapse or disability; higher mortality risk was linked to age, comorbidities, and rituximab use. [41]. The absence of deaths in our study is likely due to the small cohort size and milder disease.

### 4.4. Limitation

This study has several limitations. First, our small, self-selected sample from a single urban tertiary institution limited generalizability, with pandemic-related travel restrictions potentially skewing participation further. Second, the cross-sectional design restricted causal inferences. Thus, the study could not capture longitudinal outcomes such as delayed relapses, treatment adherence, or quality-of-life changes. Future longitudinal studies are needed to explore these dimensions. Future longitudinal studies are warranted to explore the lasting effects of pandemics on disease trajectory, treatment adherence, and quality of life among MS patients. Third, we did not assess the impact of COVID-19 on the availability of rehabilitation and home care services for PwMS. A related limitation is that the temporal relationship between COVID-19 infection and the onset of MS was not analyzed. Fourth, the study did not explore how the pandemic affected individuals in different occupations, despite the relevance of employment status to psychological health and financial stability. Fourth, the study did not explore how the COVID-19 pandemic affected different occupations despite the links between occupational status, psychological health, and financial stability. Fifth, we did not include pre-existing chronic conditions and how they impact the trajectories of MS. Sixth, the study was not designed to explore the complex interplay of psychosocial, environmental, and national-level contextual factors related to the pandemic. Finally, this study was limited to a single country. Expanding or replicating this study in other geographic regions would offer important comparative insights, given the varied experiences of PwMS during the pandemic across healthcare systems and sociocultural settings. While such replication is difficult due to the rarity of global events like COVID-19, international collaboration could help build preparedness for interventions for PwMS in future health crises.

## 5. Conclusions

The key predictors of self-reported effects of COVID-19 among Omani PwMS were COVID-19 diagnosis, female sex, younger age, and low current mental well-being. These findings highlight the need to study the mental resilience of these subgroups of PwMS and provide them with targeted support during crises. In addition, they underscore the importance of culturally tailored mental health interventions and crisis preparation strategies in the Arabian Gulf region to address the psychosocial burden of pandemics on PwMS.

## Figures and Tables

**Table 1:** Demographic and clinical characteristics of Omani PwMS during the COVID-19 pandemic.

Variables	Overall (n = 104)	COVID-19 Uninfected (n = 81)	COVID-19 Infected (n = 23)
	n (%)	n (%)	n (%)
Sex Male	28 (26.9)	17 (21.0)	11 (47.8)
Sex, Female	76 (73.1)	64 (79.0)	12 (52.2)
Age in years, Mean (SD)	39.2 (8.5)	39.3 (7.5)	38.9 (7.5)
Duration of disease in years, Mean (SD)	12.7 (5.9)	13.2 (5.8)	10.9 (6.1)
EDSS, Mean (SD)	4.5 (4.5)	4.5 (4.6)	4.6 (4.4)
EDSS severity (disability) scores:			
No disability (score 0)	0 (0.0)	0 (0.0)	0 (0.0)
Slight (1.0–1.5)	35 (33.7)	28 (34.6)	7 (30.4)
Minimal (2.0–2.5)	19 (18.3)	15 (18.5)	4 (17.4)
Moderate (3.0–4.5)	19 (18.3)	14 (17.3)	5 (21.7)
Severe (≥5.0)	30 (28.8)	23 (28.4)	7 (30.4)
Use of DMTs			
Any DMT	88 (84.6)	67 (82.7)	21 (91.3)
None	16 (15.4)	14 (17.3)	2 (8.7)
Oral DMT (teriflunomide, fingolimod, dimethyl fumarate)	38 (36.5)	26 (32.1)	12 (52.2)
Oral IRT (reconstitution therapy: cladribine)	4 (3.8)	4 (4.9)	0 (0.0)
DMT infusion (ocrelizumab, rituximab, natalizumab)	41 (39.4)	32 (39.5)	9 (39.1)
Injectable DMT (interferons and glatiramer acetate)	5 (4.8)	5 (6.2)	0 (0.0)
Effect of the COVID-19 pandemic period			
Continued to take DMT	94 (90.4)	76 (93.8)	18 (78.3)
MS relapse(s) occurred	13 (12.5)	9 (11.1)	4 (17.4)
Received IV methylprednisolone for relapse	10 (9.6)	8 (9.9)	2 (8.7)
Had problems getting prescriptions	5 (4.8)	3 (3.7)	2 (8.7)
Neurologist appointments affected	3 (2.9)	3 (3.7)	0 (0.0)
MRI appointment affected	20 (19.2)	15 (18.5)	5 (21.7)

Note: MS: multiple sclerosis; PwMS: people with multiple sclerosis; EDSS: Expanded Disability Status Scale; DMT: disease-modifying therapy; IV: intravenous; MRI: magnetic resonance imaging.

**Table 2:** Distribution of symptoms and other variables related to Omani PwMS who developed COVID-19 infection (n = 23).

Variable	Total Patients (n = 23)	Male (n = 11)	Female (n = 12)	Odds Ratio (OR)	*p*-Value
	n (%)	n (%)	n (%)		
Fatigue	20 (87.0%)	9 (81.8)	11 (91.7)	0.41	0.59
Loss of taste	18 (78.3%)	10 (90.9)	8 (66.7)	5.00	0.32
Fever	17 (73.9%)	8 (72.7)	9 (75.0)	0.89	1.00
Headache	16 (69.6%)	8 (72.7)	8 (66.7)	1.33	1.00
Chest pain	13 (56.5%)	6 (54.5)	7 (58.3)	0.86	1.00
Shortness of breath	11 (47.8%)	4 (36.4)	7 (58.3)	0.41	0.41
Dry cough	12 (52.2%)	5 (45.5)	7 (58.3)	0.60	0.68
Sore throat	12 (52.2%)	5 (45.5)	7 (58.3)	0.60	0.68
Diarrheal	2 (8.7%)	2 (18.2)	0 (0.0)	inf	0.22
Redness of eyes	1 (4.3%)	0 (0.0)	1 (8.3)	0.00	1.00
Patient was managed at home	21 (91.3%)	10 (90.9)	11 (91.7)	0.91	1.00
Fully recovered from COVID-19	17 (73.9%)	9 (81.8)	8 (66.7)	2.25	0.64
COVID-19 had no lasting effect on MS	18 (78.3%)	10 (90.9)	8 (66.7)	5.00	0.32
MS symptoms worsened by COVID-19	1 (4.3%)	0 (0.0)	1 (8.3)	0.00	1.00

Note: MS: multiple sclerosis; PwMS: people with multiple sclerosis.

**Table 3:** Psychological concerns and experiences of Omani patients with multiple sclerosis during the COVID-19 pandemic period (n = 104).

Characteristic	Response	Overall Sample (n = 104)	Male (n = 28)	Female (n = 76)	Chi-Square	*p*-Value
		n (%)	n (%)	n (%)		
COVID-19 infection worries	All the time	48 (46.2)	18 (64.3)	30 (39.5)	5.98	0.20
	Most of the time	21 (20.2)	5 (17.9)	16 (21.1)		
	Sometimes	24 (23.1)	3 (10.7)	21 (27.6)		
	Rarely	10 (9.6)	2 (7.1)	8 (10.5)		
	Never	1 (1.0)	0 (0.0)	1 (1.3)		
Healthcare access worry	All the time	54 (51.9))	18 (64.3)	36 (47.4)	3.54	0.47
	Most of the time	24 (23.1)	6 (21.4)	18 (23.7)		
	Sometimes	17 (16.3)	2 (7.1)	15 (19.7)		
	Rarely	8 (7.7)	2 (7.1)	6 (7.9)		
	Never	1 (1.0)	0 (0.0)	1 (1.3)		
MS relapse worry	All the time	50 (48.1)	17 (60.7)	33 (43.4)	6.28	0.10
	Most of the time	21 (20.2)	7 (25.0)	14 (18.4)		
	Sometimes	25 (24.0)	2 (7.1)	23 (30.3)		
	Rarely	8 (7.7)	2 (7.1)	6 (7.9)		

**Table 4:** Logistic regression model for the COVID-19 effects reported by Omani people with multiple sclerosis during the COVID-19 pandemic (n = 104).

Variable	Category	Effect Not Reported n = 48 (%)	Effect Reported n = 56 (%)	Unadjusted OR (95% CI, *p*-Value)	Adjusted OR () (95% CI, *p*-Value)
Sex	male ^†^	18 (64.3)	10 (35.7)	1.00	1.00
	female	30 (39.5)	46 (60.5)	2.76 (1.14–6.99, *p* = 0.027 *)	3.17 (1.04–9.61, *p* = 0.042 *)
Age group (years)	18–34 ^†^	5 (31.2)	11 (68.8)	1.00	1.00
	35–45	35 (55.6)	28 (44.4)	0.36 (0.10–1.12, *p* = 0.090)	0.18 (0.05–0.71, *p* = 0.014 *)
	>45	8 (32.0)	17 (68.0)	0.97 (0.24–3.70, *p* = 0.960)	0.65 (0.14–2.95, *p* = 0.573)
Diagnosed with COVID-19	No ^†^	37 (45.7)	44 (54.3)	1.00	1.00
	Yes	11 (47.8)	12 (52.2)	0.92 (0.36–2.35, *p* = 0.855)	5.76 (1.11–29.85, *p* = 0.037 *)
Chest pain	No ^†^	40 (44.0)	51 (56.0)	1.00	1.00
	Yes	8 (61.5)	5 (38.5)	0.49 (0.14–1.58, *p* = 0.241)	0.14 (0.02–1.04, *p* = 0.055)
MS relapse	No ^†^	42 (46.2)	49 (53.8)	1	1
	Yes	6 (46.2)	7 (53.8)	1.00 (0.31–3.33, *p* = 1.000)	0.68 (0.16–2.82, *p* = 0.597)
Current mental well-being Mean ± SD		19.8 ± 4.1	17.4 ± 4.7	0.88 (0.78–0.96, *p* = 0.010 *)	0.88 (0.79–0.98, *p* = 0.021 *)

* Significant at 0.05 alpha level; ^†^ reference category.

## Data Availability

Data is unavailable due to privacy.

## Abbreviations

The following abbreviations are used in this manuscript:

COVID-19	Coronavirus disease 2019
DMTs	Disease-modifying therapies
EDSS	Expanded Disability Status Scale
MENACTRIMS	Middle East North Africa Committee for Treatment and Research in MS
MOH	Ministry of Health, Oman
MS	Multiple sclerosis
PPMS	Primary progressive MS
PwMS	People with MS
RRMS	Relapsing-remitting MS
SPMS	Secondary progressive MS
SQUH	Sultan Qaboos University Hospital
U.S.	United States
UAE	United Arab Emirates
WHO	World Health Organization
WHO-5	World Health Organization Well-being Index

## References

[1] World Health Organization (2020). Coronavirus Disease (COVID-19). https://www.who.int/news-room/fact-sheets/detail/coronavirus-disease-(covid-19).

[2] Al-Senani M., Al-Salti A., Nandhagopal R., Al-Zakwani I., Alkhabouri J., Elyas M.E., Gujjar A.R., Al-Asmi A. (2021). Epidemiology of multiple sclerosis in the Sultanate of Oman: A hospital-based study. Mult. Scler. Relat. Disord..

[3] Zheng C., Kar I., Chen C.K., Sau C., Woodson S., Serra A., Abboud H. (2020). Multiple Sclerosis Disease-Modifying Therapy and the COVID-19 Pandemic: Implications on the Risk of Infection and Future Vaccination. CNS Drugs.

[4] Brownlee W., Bourdette D., Broadley S., Killestein J., Ciccarelli O. (2020). Treating multiple sclerosis and neuromyelitis optica spectrum disorder during the COVID-19 pandemic. Neurology.

[5] Wang C., Pan R., Wan X., Tan Y., Xu L., McIntyre R.S., Choo F.N., Tran B., Ho R., Sharma V.K. (2020). A longitudinal study on the mental health of general population during the COVID-19 epidemic in China. Brain Behav. Immun..

[6] Wang C., Pan R., Wan X., Tan Y., Xu L., Ho C.S., Ho R.C. (2020). Immediate Psychological Responses and Associated Factors during the Initial Stage of the 2019 Coronavirus Disease (COVID-19) Epidemic among the General Population in China. Int. J. Environ. Res. Public Health.

[7] Al-Asmi A., Al-Rawahi S., Al-Moqbali Z.S., Al-Farsi Y., Essa M.M., El-Bouri M., Koshy R.P., Gujjar A.R., Jacob P., Al-Hodar A. (2015). Magnitude and concurrence of anxiety and depression among attendees with multiple sclerosis at a tertiary care Hospital in Oman. BMC Neurol..

[8] Özdin S., Bayrak Özdin Ş. (2020). Levels and predictors of anxiety, depression and health anxiety during COVID-19 pandemic in Turkish society: The importance of gender. Int. J. Soc. Psychiatry.

[9] Worldometer (2021). Real-Time World Statistics: Corona Update Live. https://www.worldometers.info/coronavirus/.

[10] Chiaravalloti N.D., Amato M.P., Brichetto G., Chataway J., Dalgas U., DeLuca J., Meza C., Moore N.B., Feys P., Filippi M. (2020). The emotional impact of the COVID-19 pandemic on individuals with progressive multiple sclerosis. J. Neurol..

[11] Thompson A.J., Banwell B.L., Barkhof F., Carroll W.M., Coetzee T., Comi G., Correale J., Fazekas F., Filippi M., Freedman M.S. (2018). Diagnosis of multiple sclerosis: 2017 revisions of the McDonald criteria. Lancet Neurol..

[12] Hollen C., Bernard J. (2022). Multiple Sclerosis Management During the COVID-19 Pandemic. Curr. Neurol. Neurosci. Rep..

[13] Mungmunpuntipantip R., Wiwanitkit V. (2023). Multiple sclerosis patients’ response to COVID-19 pandemic and vaccination: Correspondence. Egypt. J. Neurol. Psychiatry Neurosurg..

[14] Etemadifar M., Sedaghat N., Aghababaee A., Kargaran P.K., Maracy M.R., Ganjalikhani-Hakemi M., Rayani M., Abhari A.P., Khorvash R., Salari M. (2021). COVID-19 and the Risk of Relapse in Multiple Sclerosis Patients: A Fight with No Bystander Effect?. Mult. Scler. Relat. Disord..

[15] Topp C.W., Østergaard S.D., Søndergaard S., Bech P. (2015). The WHO-5 Well-Being Index: A systematic review of the literature. Psychother. Psychosom..

[16] Tleyjeh I.M., Saddik B., Ramakrishnan R.K., AlSwaidan N., AlAnazi A., Alhazmi D., Aloufi A., AlSumait F., Berbari E.F., Halwani R. (2022). Long term predictors of breathlessness, exercise intolerance, chronic fatigue and well-being in hospitalized patients with COVID-19: A cohort study with 4 months median follow-up. J. Infect. Public. Health.

[17] R Core Team (2022). R: A Language and Environment for Statistical Computing (Version 4.2.3).

[18] Alroughani R., Inshasi J., Al-Hashel J., Alkhaboury J., Alsalti A., Al Suwaidi R., Hassino L.H., Ahmed S.F. (2022). Prevalence, Severity, Outcomes, and Risk Factors of COVID-19 in Multiple Sclerosis: An Observational Study in the Middle East. J. Clin. Neurosci..

[19] Sormani M.P. (2020). An Italian Programme for COVID-19 Infection in Multiple Sclerosis. Lancet Neurol..

[20] Worldometer (2021). WHO Director-General’s Opening Remarks at the Media Briefing on COVID-19—March 2020. Worldometer Real-Time World Statistics: Corona Update Live..

[21] Loonstra F.C., Hoitsma E., van Kempen Z., Killestein J., Mostert J. (2020). COVID-19 in Multiple Sclerosis: The Dutch Experience. Mult. Scler..

[22] Louapre C., Collongues N., Stankoff B., Giannesini C., Papeix C., Bensa C., Deschamps R., Créange A., Wahab A., Pelletier J. (2020). Clinical Characteristics and Outcomes in Patients with Coronavirus Disease 2019 and Multiple Sclerosis. JAMA Neurol..

[23] Zeineddine M., Al-Hajje A., Salameh P., Massouh J., Saab G., Al-Roughani R., Ahmed S.F., Al-Mahdawi A., Shalaby N., Inshasi J. (2024). Disease-modifying therapies, outcomes, risk factors and severity of COVID-19 in multiple sclerosis: A MENACTRIMS registry based study. Mult. Scler. Relat. Disord..

[24] Czarnowska A., Brola W., Zajkowska O., Rusek S., Adamczyk-Sowa M., Kubicka-Bączyk K., Kalinowska-Łyszczarz A., Kania K., Słowik A., Wnuk M. (2021). Clinical Course and Outcome of SARS-CoV-2 Infection in Multiple Sclerosis Patients Treated with Disease-Modifying Therapies—The Polish Experience. Neurol. Neurochir. Pol..

[25] Abou-Rass Z., Feldpausch J., Plummer P., Fritz N.E. (2024). The Impact of COVID-19 on Fatigue in Multiple Sclerosis. Int. J. MS Care.

[26] Bellucci G., Rinaldi V., Buscarinu M.C., Reniè R., Bigi R., Pellicciari G., Morena E., Romano C., Marrone A., Mechelli R. (2021). Changes in the Health Care of People with Multiple Sclerosis from Latin America during the COVID-19 Pandemic. Mult. Scler. Relat. Disord..

[27] Zhang Y., Staker E., Cutter G., Krieger S., Miller A.E. (2021). Perceptions of Risk and Adherence to Care in MS Patients during the COVID-19 Pandemic: A Cross-Sectional Study. Mult. Scler. Relat. Disord..

[28] Alshamrani F., Alnajashi H., AlJumah M., Almuaigel M., Almalik Y., Makkawi S., Alsalman S., Almejally M., Qureshi S., Aljarallah S. (2021). Registry of Patients with Multiple Sclerosis and COVID-19 Infection in Saudi Arabia. Mult. Scler. Relat. Disord..

[29] Chaudhry F., Bulka H., Rathnam A.S., Said O.M., Lin J., Lorigan H., Bernitsas E., Rube J., Korzeniewski S.J., Memon A.B. (2020). COVID-19 in Multiple Sclerosis Patients and Risk Factors for Severe Infection. J. Neurol. Sci..

[30] Lal A.P., Foong Y.C., Sanfilippo P.G., Spelman T., Rath L., Levitz D., Fabis-Pedrini M., Foschi M., Habek M., Kalincik T. (2024). A multi-centre longitudinal study analysing multiple sclerosis disease-modifying therapy prescribing patterns during the COVID-19 pandemic. J. Neurol..

[31] Ozamiz-Etxebarria N., Dosil-Santamaria M., Picaza-Gorrochategui M., Idoiaga-Mondragon N. (2020). Stress, anxiety, and depression levels in the initial stage of the COVID-19 outbreak in a population sample in the northern Spain. Cad. Saude Publica.

[32] Alkhamees A.A., Alrashed S.A., Alzunaydi A.A., Almohimeed A.S., Aljohani M.S. (2020). The Psychological Impact of COVID-19 Pandemic on the General Population of Saudi Arabia. Compr. Psychiatry.

[33] Moghanibashi-Mansourieh A. (2020). Assessing the Anxiety Level of Iranian General Population during COVID-19 Outbreak. Asian J. Psychiatr..

[34] The Multiple Sclerosis International Federation (2023). Atlas of MS, 3rd Edition—Epidemiology Report. https://www.msif.org/wp-content/uploads/2020/10/Atlas-3rd-Edition-Epidemiology-report-EN-updated-30-9-20.pdf.

[35] Sinawi H.A., Al Balushi N., Al-Mahrouqi T., Al Ghailani A., McCall R.K., Sultan A., Al Sabti H., Al Maniri A., Murthi Panchatcharam S., Al-Alawi M. (2021). Predictors of Psychological Distress among the Public in Oman amid Coronavirus Disease 2019 Pandemic: A Cross-Sectional Analytical Study. Psychol. Health Med..

[36] Vogel A.C., Schmidt H., Loud S., McBurney R., Mateen F.J. (2020). Impact of the COVID-19 Pandemic on the Health Care of >1,000 People Living with Multiple Sclerosis: A Cross-Sectional Study. Mult. Scler. Relat. Disord..

[37] Yeni K., Tulek Z., Terzi M. (2022). A year with the fear of COVID-19 in multiple sclerosis patients: Examination of depression, sleep quality and quality of life before and after the pandemic. Mult. Scler. Relat. Disord..

[38] Maciej W., Koper M., Gabryelski J., Brola W., Tasiemski T. (2022). Mental health status of people with multiple sclerosis during the COVID-19 pandemic. J. Clin. Med..

[39] Longinetti E., Bower H., McKay K.A., Englund S., Burman J., Fink K., Fogdell-Hahn A., Gunnarsson M., Hillert J., Langer-Gould A. (2022). COVID-19 clinical outcomes and DMT of MS patients and population-based controls. Ann. Clin. Transl. Neurol..

[40] Seyedmirzaei H., Salabat D., KamaliZonouzi S., Teixeira A.L., Rezaei N. (2024). Risk of MS relapse and deterioration after COVID-19: A systematic review and meta-analysis. Mult. Scler. Relat. Disord..

[41] Prosperini L., Tortorella C., Haggiag S., Ruggieri S., Galgani S., Gasperini C. (2022). Determinants of COVID-19-related lethality in multiple sclerosis: A meta-regression of observational studies. J. Neurol..

